# Scaling of shoot and root respiration of woody and herbaceous plants

**DOI:** 10.1098/rspb.2024.1910

**Published:** 2025-01-29

**Authors:** Yoko Kurosawa, Shigeta Mori, Juan P. Ferrio, Tomohiro Nishizono, Oxana V. Masyagina, Keiko Yamaji, Kohei Koyama, Toshikatsu Haruma, Kohei Doyama, Tomoki Hoshino, Hideki Murayama, Mitsuharu Yagi, Masayuki Yokozawa, Shingo Tomiyama

**Affiliations:** ^1^ Faculty of Agriculture, Yamagata University, Tsuruoka 997-8555, Japan; ^2^ Graduate School of Life and Environmental Sciences, University of Tsukuba, Tsukuba 305-8572, Japan; ^3^ Estación Experimental de Aula Dei (EEAD), CSIC, Zaragoza 50059, Spain; ^4^ Department of Forest Management, Forestry and Forest Products Research Institute, Tsukuba 305-8687, Japan; ^5^ Sukachev Institute of Forest SB RAS, Federal Research Center 'Krasnoyarsk Science Center SB RAS', Krasnoyarsk 660036, Russia; ^6^ Faculty of Education, Hokkaido University of Education, Asahikawa Campus, Hokumoncho, Asahikawa 070-8621, Japan; ^7^ Department of Mushroom Science and Forest Microbiology, Forestry and Forest Products Research Institute, Tsukuba 305-8687, Japan; ^8^ Research Institute for Geo-Resources and Environment, Geological Survey of Japan, National Institute of Advanced Industrial Science and Technology (AIST), Tsukuba 305-8567, Japan; ^9^ Institute of Integrated Science and Technology, Nagasaki University, Nagasaki 852-8521, Japan; ^10^ Faculty of Human Sciences, Waseda University, Tokorozawa 359-1192, Japan; ^11^ Faculty of Engineering, Hokkaido University, Sapporo 060-8628, Japan

**Keywords:** metabolic scaling, allometry, whole-plant respiration, shoot and root respiration, woody and herbaceous plants

## Abstract

Woody and herbaceous plants are the main components of global terrestrial ecosystems, and their growth, adaptation and survival depend largely on the metabolism of shoots and roots. Therefore, understanding size-scaling of metabolic rates in woody and herbaceous plants, and in shoots and roots, is a fundamental issue in ecology. However, few empirical studies have examined metabolic scaling exponents across a wide range of plant sizes. Using whole-plant chamber systems, we measured respiration rates of entire root systems and shoots of 96 woody species (*n* = 1243) and 33 herbaceous species (*n* = 463) from various terrestrial biomes, with plant masses spanning nine orders of magnitude. Scaling exponents for relationships between respiration rates and fresh mass were greater in shoots than in roots, and both were greater in herbaceous plants than in woody plants. Furthermore, scaling of whole-plant respiration, including various species, converged separately for woody and herbaceous plants. These findings suggest some general physico-chemical constraints on energy use by shoots and roots of individual plants in various terrestrial biomes, including forests and grasslands. These data will advance our understanding of terrestrial ecosystem structure and function.

## 1. Introduction

Plant respiration provides energy for carbon acquisition by shoots and water acquisition by roots [1–3] and it is crucial for carbon cycling in various vegetation types of terrestrial ecosystems, such as forests and grasslands [4–6]. Whole-plant, shoot and root respiration vary according to plant size and environmental conditions [1,6], and how they scale with plant size in various biomes is a fundamental issue in ecology that links individual metabolic rates to terrestrial carbon cycles [7–11]. Furthermore, understanding differences in respiratory scaling among plant functional types, e.g. evergreen versus deciduous, annual versus biennial versus perennial—especially those based on growth forms, i.e. woody versus herbaceous plants—should promote integrated understanding of carbon cycling among terrestrial biomes and enable simplification of models of vegetation dynamics in terrestrial ecosystems [5,12–17]. However, few empirical studies have measured whole-plant respiration rates across a wide range of herbaceous and woody plant sizes [6]. Especially for large trees, respiration measurements of entire root systems have been rare because they involve many difficult, time-consuming tasks [6,10]. For these reasons, differences in scaling of shoot and root respiration according to plant growth forms are still poorly understood.

Scaling of individual metabolic rates with organismal size has usually been described using an allometric equation, or a simple power function of the form


(1.1) $$Y={FM}^{f},$$

where *M* is the individual fresh mass, *Y* is the individual respiration rate, *F* is an intercept and *f* is the scaling exponent (the slope of log–log coordinates) [1,18–22]. Since Kleiber [18] empirically observed simple and robust allometry of *f* = 0.75, most theoretical work has focused on the value of *f* and has attempted to explain metabolic scaling relationships. West *et al*. [19] proposed a model unifying metabolic rates for plants and animals and predicted that *f* would converge to 0.75. This is now called the ‘metabolic theory of ecology’. Actually, metabolic scaling has been reported to show similarity and tight convergence across widely different life forms, including both plants and animals [21,22]. The existence of similar scaling among divergent organisms is unlikely to occur by chance alone, suggesting that convergent evolution in relation to energy and material transport has been caused partly by general physico-chemical constraints, e.g. gravitational force [23,24]. Convergence in metabolic scaling would provide evidence that organisms of divergent life forms have evolved to achieve equivalent energetic efficiencies [21,22]. However, the value of exponent *f* for terrestrial plants remains controversial [22,25,26], since few empirical studies have tested whole-plant respiration rates covering a wide range of plant sizes from seedlings to larger trees. Furthermore, few empirical studies have tested differences in *f* between shoots and roots, and they have either focused only on one species [27,28] or obtained most of their data from plants grown under controlled greenhouse conditions [29]. In metabolic scaling theory, the exponent *f* has been assumed to converge over diverse species and environments [23,24,30]. In this context, empirical metabolic scaling encompassing diverse plant functional types and biomes would provide insight into such general constraints on terrestrial plants as they relate to energy use in shoots and roots, and would help us to understand the terrestrial carbon cycle with a broader perspective [6].

**Figure 1.**
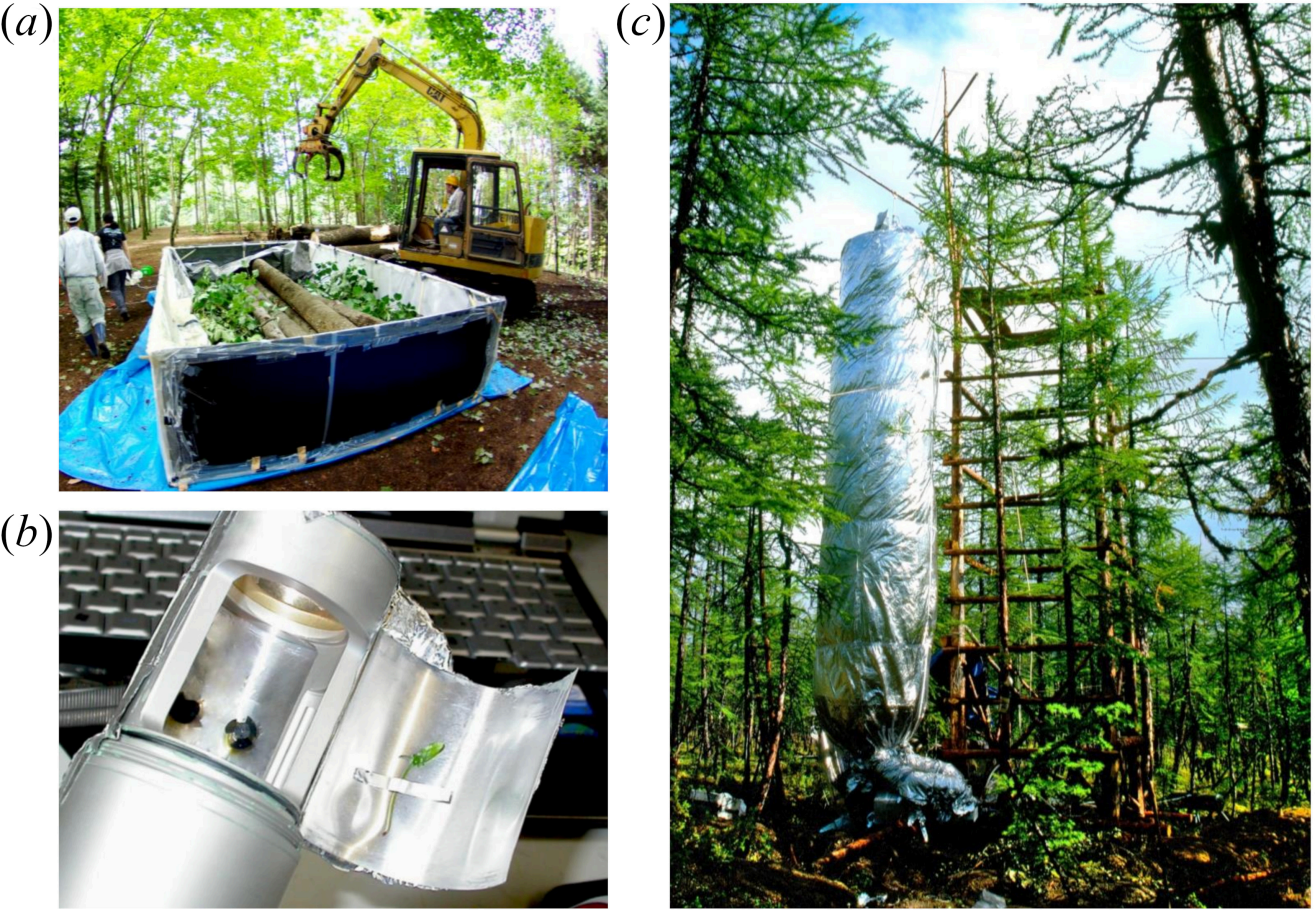
Methods for measuring whole-plant respiration from seedlings to giant trees using closed-air circulation chambers for all sample plants. (*a*) Measurement of excised and excavated materials from a large tree using a destructive method. (*b*) Measurement of excised or excavated seedlings. (*c*) Measurement of a standing tree using a dark chamber in Siberia, with air temperature control. These methods were developed by Mori *et al*. [31,32].

Mori *et al*. [31,32] developed whole-plant chamber methods that enable efficient, accurate measurement of whole-plant respiration (figure 1) and measured whole-plant respiration rates of woody species from various biomes. They found that the exponent *f* for whole-plant respiration was slightly over 1.0 in the smallest plants and gradually decreased to below 1.0 in large trees [31]. Kurosawa *et al*. [27] and Cheng *et al*. [33] also empirically showed similar ontogenetic changes in exponent *f* for plants. Ontogenetic changes in *f* have been also reported for animals and other organisms [20,21,26,34–36], and are thought to be explained by changes in body composition and cell size [26]. For plants, the ontogenetic decrease of *f* should result partly from to continuous accumulation of metabolically inert (woody) tissues in stems and roots owing to secondary growth [26,31,37–39]. Indeed, several empirical studies that removed effects of metabolically inactive tissue by correcting for water content [29] or that evaluated relationships between metabolic rates and nitrogen content [40] reported that *f* was approximately 1.0, regardless of body size or ontogenetic stage. Secondary growth is pronounced for woody plants and marginal for herbaceous plants. Therefore, comparisons of metabolic rates between woody and herbaceous plants would help to explain how changes in the proportion of tissues are related to ontogenetic changes in *f* in terrestrial plants.

In the present study, we used whole-plant chambers to compile shoot and root respiration data from various functional types of woody and herbaceous plants from different environments to cover an array of factors affecting metabolic rates[31] (figure 1). It is generally accepted that plant respiration rates in a specific environment reflect both active (plastic) responses to environmental factors and developmentally fixed aspects of plant growth [41–43]. Therefore, we adopted an approach of sampling of plants from diverse environmental conditions in biomes from tropical to subpolar zones, which is advantageous in both understanding scaling and considering potential variation of metabolic rates owing to plasticity in response to environmental factors. The resulting database spanned nine orders of magnitude in whole-plant mass (3.40 × 10^−6^−10 900 kg), or almost the full range of terrestrial plant sizes in nature. This database allowed us to undertake the most comprehensive analysis of the scaling of shoot and root respiration rates to date. Using this dataset, we tested differences in metabolic scaling between woody and herbaceous plants, and among different functional types (i.e. deciduous versus evergreen species for woody plants; annual versus biennial versus perennial species for herbaceous plants) and lineages (i.e. angiosperms versus gymnosperms for woody plants; monocots versus eudicots for herbaceous plants). In the present study, we focus on how metabolic scaling in shoots and roots converges (or diverges) among various plant functional types across terrestrial biomes.

## 2. Material and methods

### (a) Plant materials and datasets

**Figure 2.**
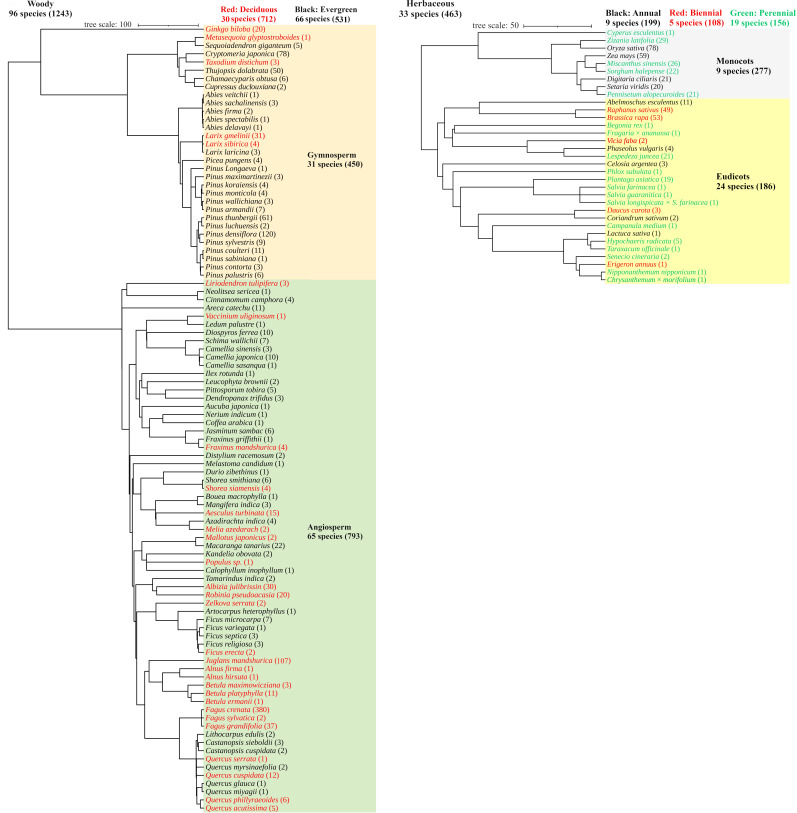
Phylogeny of the 96 woody species and 33 herbaceous species analysed in the present study. Numbers in parentheses indicate numbers of individuals. The phylogenetic tree was visualized using the Interactive Tree of Life v. 6 [44].

We compiled an extensive database of whole-plant respiration, and respiration of entire shoot and root systems of various plants growing under natural and semi-natural conditions. The database includes 96 woody species (*n* = 1243) and 33 herbaceous species (*n* = 463) (figure 2) of various biomes from tropical to subpolar zones. Plants ranged from 1 to 3400 cm in height and spanned nine orders of magnitude in whole-plant fresh mass, including roots. Data were taken from datasets of Kurosawa *et al*. [27] and Mori *et al*. [31] and additional measurements were performed during the present study on 73 species (40 woody species, *n* = 662; 33 herbaceous species, *n* = 463), all of which were collected using whole-plant chamber methods developed by Mori *et al*. [31,32] (figure 1). In these studies, sampling of plants was conducted under various environmental conditions, and included dominant trees under sufficient light and suppressed trees under restricted light. Therefore, the present database covers a wide range of respiration rates determined by various internal and environmental factors [41–43]. We used data from individual plants, with clearly separated roots, to avoid ambiguous interpretations of individual plant size. Since we sought to understand respiration scaling considering the balance between carbon acquisition by (photosynthetic) true leaves and water uptake by roots, we excluded individuals in early growth stages before the development of the first true leaves.

### (b) Measurements

Measurements of respiration rates were conducted during the growing season, or spring to summer, using two closed-chamber methods developed by Mori *et al*. [31,32]. One was designed to measure respiration rates of excised shoots and excavated root systems (figure 1*a*,*b*, details described below). Another was designed to measure respiration rates of intact shoots of standing plants (figure 1*c*, details in [31,32]). Using these two methods, it has been confirmed that there are no differences between shoot respiration rates of excised materials and standing plants [31].

During this study, we measured the respiration of excised shoots and excavated root systems using methods shown in figure 1*a*,*b*. For both woody and herbaceous plants, whole bodies of individuals were carefully dug to a depth at which the absence of roots was confirmed. Root systems of large trees were excavated to an area over twice the size of the canopy area using a grapple on heavy equipment. Roots left in the soil were finally carefully collected using shovels. After excavation, we removed any soil on the roots by washing, cut the individuals into shoots and roots, and measured their fresh masses. Digital balances were used to measure shoot and root fresh masses for herbaceous plants and small trees, and a digital crane scale with a capacity of 2000 kg (Handy Cosmo II, Shuzui, Nagoya, Japan) was used for large trees. After fresh masses were measured, plants were covered with wet cloths to prevent transpiration and kept in a cool, dark place until the measurement of their respiration rates [17,27,31].

Plant materials were enclosed in customized incubation chambers (figure 1*a*,*b*) and CO_2_ concentrations in the chambers were measured every 5 s for 30−900 s with a CO_2_ probe (GMP343, Vaisala, Helsinki, Finland). During measurements, CO_2_ concentrations in the chamber were homogenized using forced air circulation with DC axial fans. To obtain accurate measurements for short periods, we prepared 10 chambers of different volumes (80 cm^3^–8 m^3^) and used them according to plant sizes. All chambers were confirmed to be sufficiently airtight by demonstrating that there was no decrease in CO_2_ concentrations over time. In large-volume chambers, sufficient air circulation was facilitated by a cylindrical air duct attached to fans. The time from excavation to the end of respiration measurement per individual was 5−30 min for herbs and small trees. For large trees, this took several hours, mainly owing to the time-consuming excavation process. During measurements, temperature variation in the chambers was recorded ±1°C. To reconcile differences in temperature among measurements, we adjusted all respiration rates to 20°C, assuming *Q*_10_ = 2 [45].

### (c) Data analysis

All statistical analyses were performed in R (v. 4.2.1, R Core Team [46]). We ran a phylogenetically controlled Markov-chain Monte Carlo (MCMC) generalized linear mixed model using the package ‘MCMCglmm’ [47]. MCMCglmm uses a Bayesian approach to fit phylogenetic generalized linear mixed models (PGLMM) and includes phylogeny as a random effect in the regression model [48]. This approach formally incorporates non-independence associated with phylogenetic relatedness as well as non-independence associated with multiple measurements of single species in the database; thus, the model includes ontogenetic scaling of various species. Assuming allometric relationships between respiration rate and fresh mass (equation (1.1)), we used slopes from linear regression fitted to our log-transformed data to estimate the scaling exponent *f*. A phylogenetic tree including all species in our database was constructed using the package ‘V.PhyloMaker2’ [49], with GBOTB phylogeny (GenBank taxa with a backbone provided by Open Tree of Life version 9.1) [50] as the backbone (figure 2). MCMCglmm analysis was performed for datasets of woody and herbaceous plants, including all species, and for sub-datasets separated by their functional types, using different phylogenetic trees according to the species in each dataset. Each analysis was based on three chains with non-informative prior 1 set to 1 28 000 iterations, along with 3000 for burn-in, and a thinning interval of 50. Convergence of chains was verified using the Gelman–Rubin statistic ($\hat{R}$ < 1.1) [51] in the package ‘CODA’ [52]. Statistical inference to compare scaling exponents was based on 95% confidence intervals (CIs) and we concluded that they were significantly different if they exhibited no overlap of 95% CIs. Here, we report statistical results for the first run of the three MCMC chains.

## 3. Results

### (a) Differences in metabolic scaling between woody and herbaceous plants

**Figure 3.**
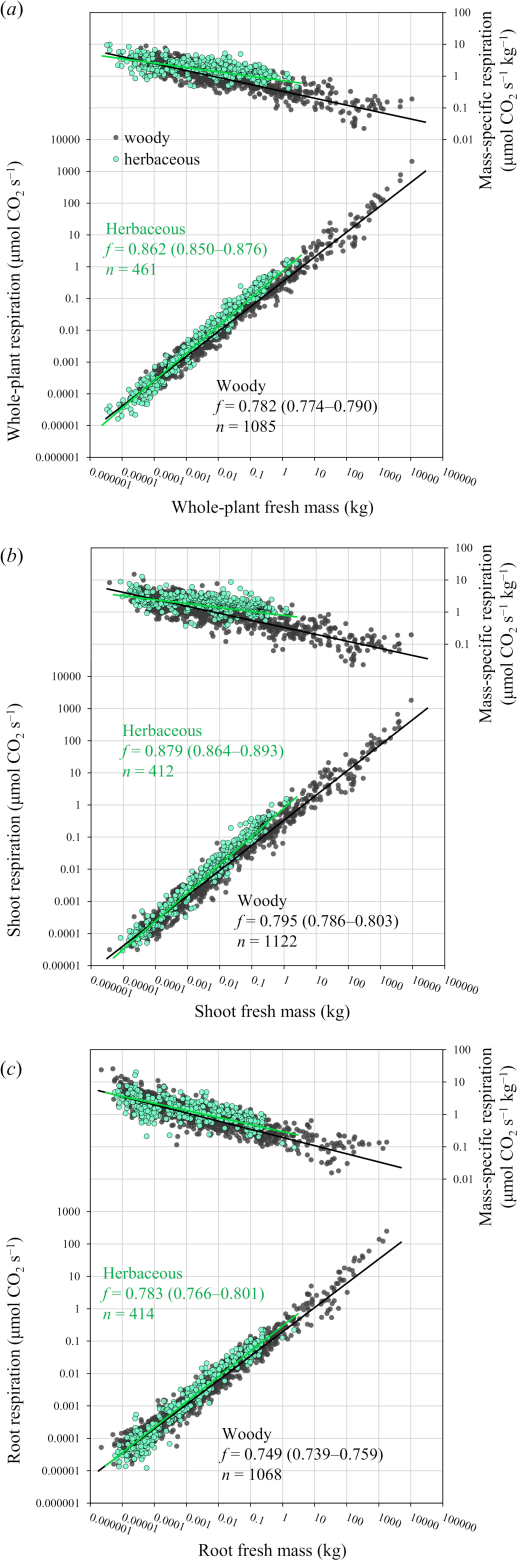
Scaling exponents *f* for relationships between respiration and fresh mass for (*a*) whole plants, (*b*) shoots and (*c*) roots are statistically higher in herbaceous plants (green) than woody plants (black). Respiration rates were adjusted to 20°C based on *Q*_10_ = 2. Lines show Bayesian PGLMM. The values in parentheses specify the 95% CI of the scaling exponent, *f*.

First, we tested relationships between respiration rate and fresh mass, using our extensive database for 96 woody species (*n* = 1243) and 33 herbaceous species (*n* = 463) (figure 3 and Table 1). For both woody and herbaceous plants, scaling exponents *f* in whole plants, shoots and roots were statistically <1.0. Exponents *f* for whole-plant respiration were *f* = 0.782 (95% CI: 0.774−0.790) in woody plants and *f* = 0.862 (95% CI: 0.850−0.876) in herbaceous plants, showing that the value was closer to 0.75 in woody plants than in herbaceous plants. Furthermore, for both woody and herbaceous plants, exponents *f* were statistically greater in shoots than in roots, and respiration rates of shoots and roots were similar at the smallest mass (electronic supplementary material, figure S1). Consequently, for both woody and herbaceous plants, mass-specific respiration decreased more in roots than in shoots as size increased, and it tended to be greater in shoots than in roots across a wide mass range (electronic supplementary material, figure S1).

**Table 1.** Summary of statistics from Bayesian PGLMM considering phylogeny, for scaling relationships between respiration andfresh mass in whole plant, shoot and root for woody and herbaceous plants.

part	growth form	number of individuals	number of species	exponent *f* (95% CI)	pMCMC	intercept *F* (95% CI)	pMCMC
whole plant	woody	1085	85	0.782 (0.774−0.790)	<0.001	0.331 (0.183−0.599)	<0.001
	herbaceous	461	33	0.862 (0.850−0.876)	<0.001	0.731 (0.455−1.20)	0.188
shoot	woody	1122	96	0.795 (0.786−0.803)	<0.001	0.331 (0.181−0.591)	<0.001
	herbaceous	412	31	0.879 (0.864−0.893)	<0.001	0.781 (0.485−1.24)	0.278
root	woody	1068	86	0.749 (0.739−0.759)	<0.001	0.194 (0.0935−0.420)	<0.001
	herbaceous	414	31	0.783 (0.766−0.801)	<0.001	0.296 (0.157−0.562)	0.00160

Exponent *f* and intercept *F* denote posterior means.

**Figure 4.**
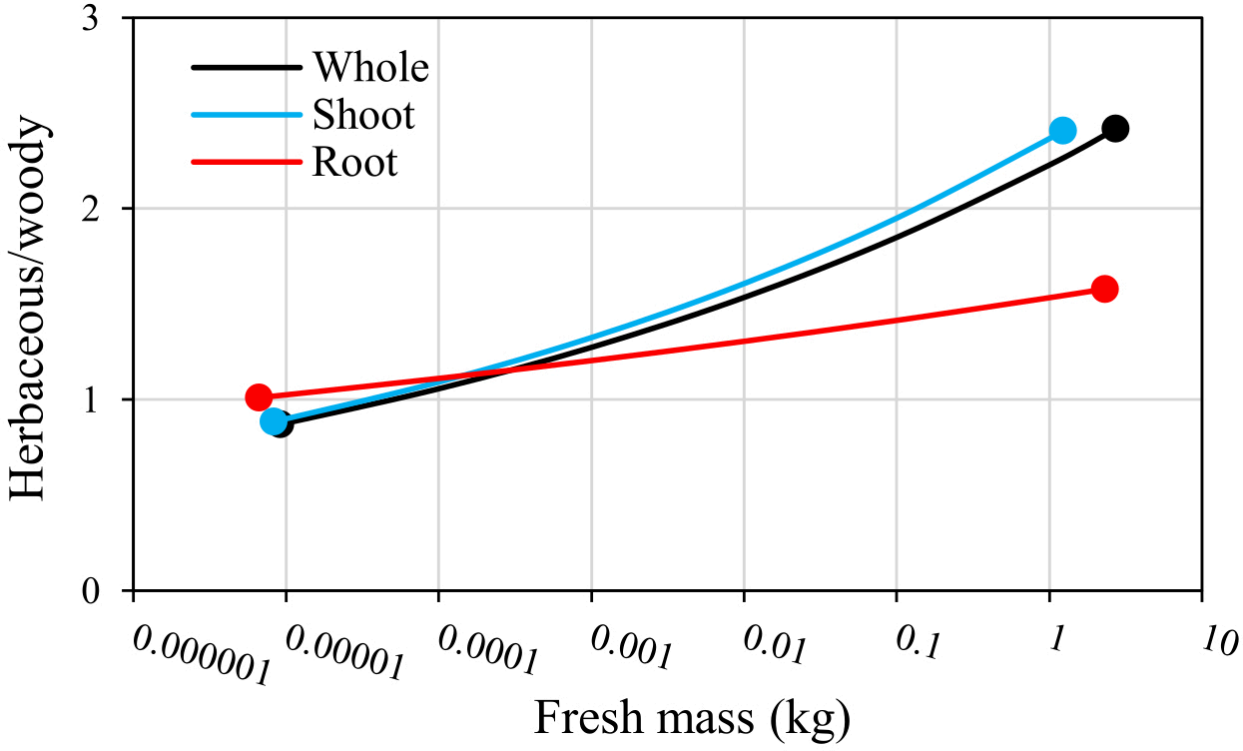
Shift of respiration rates according to fresh mass in herbaceous and woody plants is more consistent (herbaceous to woody plant ratio is closer to 1.0 over a wide mass range) in roots (red) rather than in shoots (blue) and whole plants (black). Herbaceous/woody ratios are calculated by Bayesian PGLMM in figure 3. Mass ranges correspond to the amount of overlap between masses of woody and herbaceous plants in the present database.

Mass-specific respiration of whole plants, shoots and roots was almost consistent between woody and herbaceous plants of the smallest mass, but diverged ontogenetically (figure 3*a*–c). This was because the decrease of mass-specific respiration with increasing mass is greater in woody plants than in herbaceous plants. However, despite increasing differences between woody and herbaceous plants with increasing mass, their respiration rates overlapped at any given mass. Further analysis of these results suggested that the increasing difference in respiration rates between woody and herbaceous plants with increasing mass is greater in shoots than in roots (figure 4). These findings indicate that respiration rates of woody and herbaceous plants overlap more in roots than in shoots over a wide mass range.

### (b) Differences in metabolic scaling among lineages and functional types in woody and herbaceous plants

**Figure 5.**
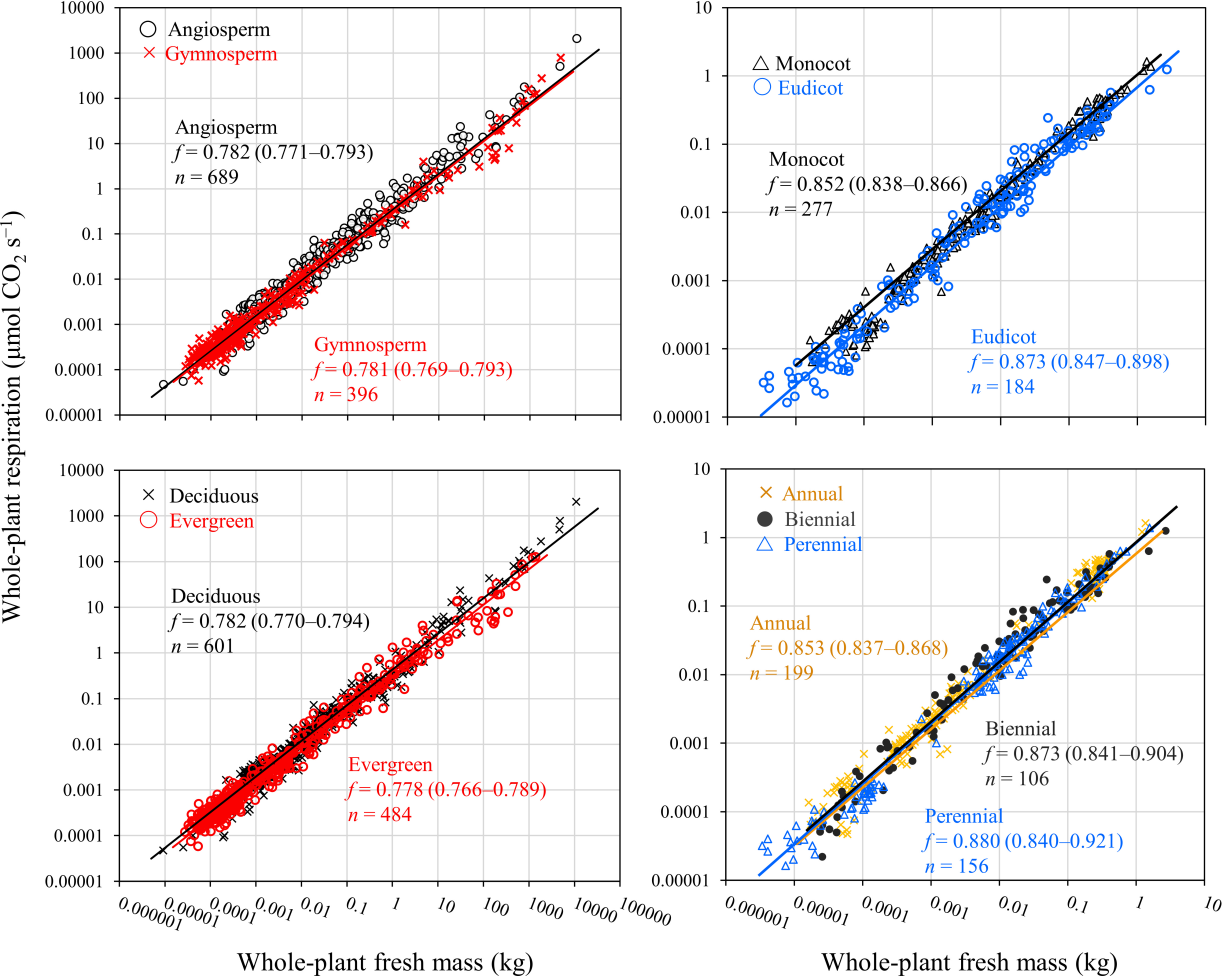
Consistent scaling of whole-plant respiration with whole-plant fresh mass among various lineages and functional types across woody and herbaceous plants. Lines show Bayesian PGLMM. Values in parentheses are 95% CIs for scaling exponent, *f*.

Subsequently, we compared the scaling of whole-plant respiration of different lineages and functional types in woody and herbaceous plants (figure 5 and table 2). For woody plants, there was no significant difference in scaling exponents between angiosperms (*n* = 689) and gymnosperms (*n* = 396), nor between deciduous (*n* = 601) and evergreen (*n* = 484) species. Similarly, for herbaceous plants, there was also no significant difference in scaling exponents between monocots (*n* = 277) and eudicots (*n* = 184), nor among annuals (*n* = 199), biennials (*n* = 106) and perennials (*n* = 156). In each case for woody and herbaceous plants, fitting lines of different functional types showed considerable consistency. Finally, we also compared scaling exponents for whole-plant respiration in woody plants among the three datasets: from Mori *et al*. [31], from Kurosawa *et al*. [27] and the dataset obtained during this work. Since the dataset from Kurosawa *et al*. [27] included only one species, we did not incorporate random effects in the fitting analysis for their dataset. There was no statistical difference in scaling exponents among the three datasets, and their fitting lines showed considerable consistency (electronic supplementary material, figure S2). Thus, our results suggest that metabolic scaling of different lineages and functional types converge strongly for woody and herbaceous plants, considered separately.

**Table 2.** Summary of statistics from Bayesian PGLMM, considering phylogeny, for scaling relationships between whole-plant respiration and whole-plant fresh mass for various lineages and functional types of woody and herbaceous plants.

growth form	group	number of individuals	number of species	exponent *f* (95% CI)	pMCMC	intercept *F* (95% CI)	pMCMC
woody	angiosperm	689	56	0.782 (0.771−0.793)	<0.001	0.349 (0.265−0.450)	<0.001
	gymnosperm	396	29	0.781 (0.769−0.793)	<0.001	0.312 (0.137−0.693)	0.0120
	deciduous	601	27	0.782 (0.770−0.794)	<0.001	0.414 (0.220−0.742)	0.0104
	evergreen	484	58	0.778 (0.766−0.789)	<0.001	0.312 (0.165−0.618)	<0.001
herbaceous	monocot	277	9	0.852 (0.838−0.866)	<0.001	1.05 (0.330−3.41)	0.934
	eudicot	184	24	0.873 (0.847−0.898)	<0.001	0.68 (0.473−0.900)	0.0200
	annual	199	9	0.853 (0.837−0.868)	<0.001	0.581 (0.376−0.829)	0.0104
	biennial	106	5	0.873 (0.841−0.904)	<0.001	0.857 (0.448−1.82)	0.593
	perennial	156	19	0.880 (0.840−0.921)	<0.001	0.836 (0.396−1.65)	0.601

Exponent *f* and intercept *F* denote posterior means.

## 4. Discussion

This study covered a wide range of sizes of plants growing under natural and semi-natural conditions and found statistical differences in exponents for scaling of respiration of whole-plants, shoots and roots between herbaceous and woody plants. As far as we are aware, this is the first empirical study to quantify differences in metabolic scaling between woody and herbaceous plants, covering various internal and environmental factors that potentially affect plant respiration. We found that both woody and herbaceous plants had statistically larger scaling exponents for shoots than for roots (figure 3 and table 1). This is consistent with previous empirical studies [27–29,53]. Kurosawa *et al*. [53] and Ferrio *et al*. [28] showed that mass-specific respiration rates in woody species remained nearly constant in leaves, whereas those of stems and roots decreased significantly with size, using relatively smaller trees (<10 kg). Their findings are consistent with the idea that leaves are responsible for higher exponents in shoots than in stems and roots [28,53]. That interpretation might also seem to be consistent with our results; we also found higher exponents for shoots than roots for both woody and herbaceous plants. However, our study included giant trees of 10 900 kg that allocated most of their biomass to woody tissues not leaves, and so leaves cannot explain higher shoot respiration rates across the range of plants included in our study. Instead, differences between shoots and roots in our results are more likely owing to differences in the rate of accumulation of inert tissues in stems and roots during ontogeny, rather than to leaves. It is also likely that root respiration tends to be negatively associated with root diameter [e.g. 54,55], but little is known about the relationship between respiration rates and structure in root systems compared to those in shoots [10]. In this regard, it is important that future empirical studies examine the distribution and gradients of respiration rates in stems and roots with the goal of explaining general differences in scaling of metabolic rates between shoots and roots. Furthermore, despite significant differences in scaling exponents between woody and herbaceous plants, their metabolic rates showed considerable overlap across a wide mass range. These results not only highlight clear differences in metabolic scaling exponents between woody and herbaceous plants but also suggest considerable convergence of metabolic rate owing to common physico-chemical constraints across various terrestrial biomes [23,24]. In individual plants, it seems that total amounts of energy available to shoots and roots for carbon gain and water acquisition, respectively, are largely equivalent between woody and herbaceous species.

Exponents for scaling of whole-plant, shoot and root respiration rates are statistically higher in smaller herbaceous plants than larger woody plants (figure 3). In addition, herbaceous plants have higher mass-specific respiration rates than woody plants across a wide range of sizes, except for the smallest masses (figure 3). These findings are largely consistent with previous reports that smaller organisms have higher mass-specific metabolic rates [18,56,57]. Woody plants decrease mass-specific respiration of stems and roots during ontogeny [27,28,53] because of their continuous secondary growth that accumulates metabolically inert tissues in stems and roots [26,31,37–39]. Secondary growth of woody plants enhances mechanical support against wind and gravity, allowing them to grow larger, but this may also slow individual growth by reducing metabolic rates. Since herbaceous plants have little or no secondary growth, they maintain higher metabolic rates in shoots and roots and higher biomass allocation to leaves during development [58]. Therefore, it is reasonable that smaller herbaceous plants have higher metabolic scaling exponents and faster growth than woody plants. These findings are partly consistent with assumptions by previous theoretical studies that metabolic rates are ultimately governed by total plant photosynthetic biomass [59]. Our results suggest that the decrease in scaling exponents from seedlings to mature trees [31,33] can be explained by the increase of metabolically inert woody tissues owing to secondary growth in stems and roots, as previous studies suggested [26,38,39]. On the other hand, for metabolic scaling of animals and plants, Glazier [26] suggested that ontogenetic changes in exponents are partly related to cell enlargement. In woody plants, cell enlargement involves increased lignification and/or formation of relatively large water-filled vacuoles that provide needed turgor pressure, both of which increase structural support. Considering that woody plants, which need strong structural support, show significantly lower scaling exponents than herbaceous plants, both explanations seem reasonable and are likely to contribute to ontogenetic changes in respiration rates. Therefore, our results help to explain how metabolic scaling in plants is related to body composition and cell sizes.

We also found that overlap of metabolic scaling between woody and herbaceous plants is greater in roots than shoots (figure 4). This seems consistent with previous studies based on global metadata analysis that compared woody and herbaceous plants in scaling relationships between respiration and nitrogen concentration in leaves, stems and roots [5,13]. For example, Atkin *et al*. [13] showed that mass-specific respiration of leaves at any given leaf nitrogen concentration is higher in herbs than in woody plants, while Han and Zhu [5] reported that mass-specific respiration of fine roots does not differ significantly between woody and herbaceous plants. Furthermore, studies of plant traits other than respiration rates based on global metadata analysis have also shown that differences between woody and herbaceous plants are greater in shoots than roots [60,61]. Therefore, it is likely that various root traits, including respiration, are more consistent than shoot traits at various levels (single leaf and fine root to entire shoot and root systems) between woody and herbaceous plants. Similarly, Mori and Hagihara [55] showed that there is greater consistency in root respiration than in stem respiration among individual trees in even-aged plantations, including dominant and suppressed trees. Hence, the greater consistency in root traits among terrestrial plants may have partly emerged from more stable environmental conditions below ground than above.

We detected remarkable convergence in metabolic scaling among different lineages and functional types in forests and grasslands of various terrestrial biomes (figure 5 and table 2). This is consistent with findings by Kurosawa *et al*. [27] that there are no significant intra-specific differences in scaling of whole-plant respiration among different provenances. It should be noted that our samples were collected from various environmental conditions; thus, our results likely reflect potential plasticity of respiration rates owing to variation in environmental conditions. How phenotypic plasticity, including in metabolic rates, differs or remains similar among functional types is one of the central, but unsolved issues in ecology [42,43]. Considering that our study covered the widest ranges in environmental conditions and body mass, our results may indicate that for both woody and herbaceous plants, there is similar plasticity in whole-plant respiration rates between divergent functional types. In addition, it is worth noting the strong consistency in metabolic scaling in spite of wide variation in leaf- and root-level traits [60,61] and biomass allocation to shoots and roots [58] among species and environments. Our results suggest that convergence of plant traits tends to emerge at the whole-plant level rather than the organ level, and convergence is more likely to be observed when we perform comprehensive plant sampling that covers potential variation of plant traits.

Enhancing our understanding of size-scaling of whole-plant respiration may help to link divergence and convergence in functional traits among various species. Our findings highlight the importance of studying size-scaling of whole-plant traits to understand functional roles in terrestrial vegetation dynamics of woody and herbaceous plants and their ecosystem-level energy flow. Finally, given that scaling exponents are significantly higher in small herbaceous plants than in large woody plants, scaling of whole-plant respiration in terrestrial plants including woody and herbaceous plants may be modelled by nonlinear, downward-concave trends, as suggested by Mori *et al*. [31]. These results also help to scale-up physiological traits from leaf-level to ecosystem-level and lead to unified understanding of the structure and function of global terrestrial ecosystems.

## Data Availability

Our data and code are available in the Dryad Digital Repository [62]. Supplementary material is available online [63].
